# The effect of heat stress on the hindgut microbiota and metabolites of Simmental heifers

**DOI:** 10.3389/fmicb.2025.1724640

**Published:** 2026-01-20

**Authors:** Liutao Gao, Zhipeng Zhang, Pengtao Li, Ziying Liu, Minjie Li, Ruoxi Li, Quanzhao Tian, Zhuo Zheng

**Affiliations:** 1 Dingyuan Cattle Breeding Co., Ltd., Zhengzhou, China; 2 College of Animal Science and Technology, Henan Agricultural University, Zhengzhou, China

**Keywords:** heat stress, high-throughput sequencing, hindgut microbiota, metabolomic, Simmental heifers

## Abstract

**Introduction:**

Heat stress adversely affects the physiological status and productive performance of cattle; however, the mechanisms underlying heat stress-induced alterations in the hindgut microbiota and its metabolic functions remain poorly understood, particularly in beef-type Simmental cattle.

**Methods:**

In this study, Simmental heifers were exposed to heat stress, and physiological parameters and blood biochemical indices were evaluated. Hindgut microbial composition was characterized using 16S rRNA gene sequencing, and metabolic profiles were analyzed using liquid chromatography-mass spectrometry (LC-MS)-based metabolomics.

**Results:**

Heat stress significantly increased respiratory rate and rectal temperature and induced marked changes in several blood parameters, including heat shock proteins. 16S rRNA sequencing revealed significant alterations in the relative abundance of multiple bacterial genera under heat stress, including Ruminococcaceae_UCG-013, Alistipes, Clostridium_sensu_stricto_1, Flavonifractor, Dorea, and Anaerovorax. Metabolomic pathway enrichment analysis identified seven significantly affected pathways: pyrimidine metabolism, biosynthesis of unsaturated fatty acids, amino sugar and nucleotide sugar metabolism, fatty acid biosynthesis, propanoate metabolism, inositol phosphate metabolism, and beta-alanine metabolism.

**Discussion:**

This study provides one of the first comprehensive characterizations of heat stress-associated alterations in the hindgut microbiota and metabolome of Simmental beef cattle. The findings suggest that heat stress disrupts the hindgut microbial ecosystem by reducing beneficial taxa and increasing potentially harmful bacteria, which may be linked to disturbances in host energy metabolism and physiological homeostasis.

## 1 Introduction

Simmental cattle originated in Switzerland and were historically recognized as a dual-purpose breed providing both milk and beef. With the intensification of breeding goals and evolving market demands, the breed has undergone functional differentiation, leading to the development of distinct dairy-type and beef-type lines. In major livestock-producing regions such as China, Europe, and North America, the beef-type Simmental strain has been widely adopted as a commercial beef breed owing to its rapid growth, favorable carcass traits, and adaptability to intensive production systems (Crews et al., 2003). However, under hot summer conditions, inherent physiological characteristics such as large body size and high metabolic heat production make beef-type Simmental cattle particularly vulnerable to heat stress, which increases their risk of productivity decline and disease susceptibility (Guzmán et al., 2023).

Heat stress refers to a non-specific physiological response that occurs when the ambient temperature exceeds the animal’s thermoregulatory capacity (Bishop-Williams et al., 2015). Beef cattle are particularly susceptible to heat stress due to their dense body hair, underdeveloped sweat glands, large body size, and high metabolic heat production. These characteristics contribute to poor heat dissipation and thermal accumulation under high-temperature conditions, ultimately disrupting thermal homeostasis and leading to elevated body temperatures and heat stress. The occurrence and severity of heat stress in beef cattle can be evaluated by monitoring environmental parameters such as ambient temperature, humidity, solar radiation, and air movement (Igono et al., 1992; Bishop-Williams et al., 2015). Additionally, physiological and biochemical indicators, such as levels of heat shock proteins (HSPs), total antioxidant capacity, and especially cortisol concentration in blood, are commonly used to assess the degree of heat stress (Magdub et al., 1982; Armstrong, 1994; Nardone et al., 1997). Among these, environmental indicators offer a more rapid, convenient, and direct means of assessment. In 1962, Bianca et al. first introduced the Temperature-Humidity Index (THI) as a tool for evaluating heat stress in dairy cattle (Bianca, 1962). Subsequent studies demonstrated a strong correlation between THI values and both milk yield and thermal comfort in Holstein cows (Johnson, 1963), laying the foundation for modern heat stress research. To date, THI remains one of the most widely used indicators for describing environmental thermal load and evaluating heat stress risk in cattle (Stefani et al., 2022). It is noteworthy that the threshold for heat stress varies considerably among different types of cattle. Dairy cows, characterized by high metabolic intensity and substantial endogenous heat production, are more sensitive to ambient temperature fluctuations. Consequently, many studies have reported that dairy cattle, due to their high metabolic heat production, begin to exhibit heat stress related responses when THI values approach approximately 68. In contrast, beef cattle generally exhibit greater heat tolerance, and their heat-stress sensitivity threshold is correspondingly higher. Multiple studies have reported that beef cattle tend to display pronounced physiological and behavioral responses to thermal load, such as increased body temperature and respiratory rate, when THI values are relatively high, often around or above 72 (Wang et al., 2025). Relevant studies in Red Angus cattle have reported that exposure to high THI conditions (approximately ≥ 72) is associated with inflammatory responses and oxidative stress–related alterations (Reith et al., 2022). Moreover, studies in Fleckvieh cattle, a dual-purpose strain within the Simmental lineage, have reported that exposure to THI values around 72 is accompanied by increases in body temperature, respiratory rate, and inflammatory responses (Amato, 2025). Therefore, THI values around 72 are often used in the literature as a reference level to describe high environmental thermal load in beef cattle, although the manifestation of heat stress ultimately depends on animal-specific physiological responses.

The gastrointestinal tract plays a critical role in digestion, nutrient absorption, and immune function, with the gut microbiota exerting profound effects on host metabolism. Increasing attention has been paid in recent years to the functions of gut microbiota, including their roles in regulating lipid metabolism, enhancing host immune responses, and modulating systemic metabolic activity (Ko et al., 2020). Under conditions of high temperature and humidity in summer, metabolic heat generated by the body cannot be dissipated effectively, leading to elevated core body temperature. This thermal stress has been reported to suppress intestinal digestive function, inhibits the activity of digestive enzymes, and reduces the metabolic energy available in the gut (Baumgart and Dignass, 2002). Previous studies have shown that heat stress decreases microbial richness and diversity in the gut, resulting in microbial dysbiosis, which in turn may impairs the digestion and absorption of dietary nutrients (Galley et al., 2014). In a healthy physiological state, intestinal microbes facilitate the digestion and absorption of dietary nutrients, contribute to amino acid synthesis, and regulate pathological metabolites and inflammatory mediators, thereby playing an important role in host energy metabolism (Gagnière et al., 2016; Felizardo et al., 2019). Moreover, gut microbiota are known to contribute to host physiology through multiple mechanisms. In general, they form a microbial barrier that supports the integrity of the intestinal epithelium, protecting against pathogenic invasion and maintaining gut homeostasis (Sansonetti, 2004). In addition, small-molecule metabolites produced by gut microbes can be absorbed by intestinal epithelial cells into the bloodstream, influencing host immune function and metabolic regulation (Putignani et al., 2016). However, these specific mechanisms were not directly assessed in the present study. Therefore, the present study aims to investigate the effects of heat stress on hindgut microbiota and their metabolic profiles in Simmental heifers using 16S rRNA sequencing and metabolomics (LC-MS) techniques. The goal is to elucidate alterations in microbial composition and associated metabolic pathways under thermal stress, thereby providing a theoretical foundation for further exploring host–microbiota interactions at the metabolic pathway level under adverse environmental conditions.

## 2 Materials and methods

### 2.1 Animal management

The animals used in this experiment were beef-type Simmental heifers. The experimental animals were primarily selected based on age and body condition, and finally, six apparently healthy Simmental heifers of similar body weight (361.2 ± 4.8 kg) and age (12.5 ± 0.4 months) were selected, which were confirmed to be non-pregnant and non-lactating. Prior to the trial, all animals underwent clinical examination by licensed veterinarians to exclude overt clinical symptoms such as fever, diarrhea, respiratory abnormalities, hoof lesions, or abnormal behaviors. The experiment was conducted at Dingyuan Cattle Breeding Co., Ltd. (Zhengzhou, China) during August and October of 2021 as NHS and HS condition for fourteen days, respectively. During the experimental period, all cows had free access to food and water, with the diet formulated according to the Chinese Meat Cattle Feeding Standard (NY/T815—2004). Detailed composition and nutrient content of the diets are presented in Table 1. All procedures involving animals were approved by the Animal Ethics Committee of Henan Agricultural University, in accordance with the Guidelines for Ethical Review of Laboratory Animal Welfare (GB/T35892).

**TABLE 1:** Feed ingredients and composition of the diets fed to experimental Simmental heifers.

Item	Content
**Ingredient (%, DM basis)**	
Whole plant corn silage	24.24
Peanut stalk	17.98
Distiller dried grains	12.18
Corn flour	14.05
Tofukasu	0.56
Concentrate feed^1^	30.99
**Nutrient composition**	
DM (%)	51.91
NEmf (MJ⋅kg^–1^)^2^	53.69
CP (%)	14.29
EE (%)	3.40
NDF (%)	34.70
ADF (%)	24.34

^1^Contains minerals and vitamins per kg concentrate,The minerals and vitamins of each kg of concentrate is as follows:VA 150∼200 k IU, VD 50∼80 k IU, VE>500 mg, Ca 8∼18%, P 2.0∼5.5%, Nacl 10∼20%, Fe 0.5∼1.5 g, Cu 0.1∼0.5 g, Zn 0.5∼2.0 g, Mn 0.4∼2.0 g, Se 5∼10 mg, I 10∼15 mg, Co 4∼10 mg.

^2^NEmf was a calculated value according to Feeding Standard of Beef Cattle, while the others were measured values. DM, dry matter; NDF, neutral detergentfiber; ADF, acid detergentfiber; CP, crude protein; EE, crude fat ether extract.

### 2.2 Sample collection and measurement

A temperature and humidity logger (Testo) was installed at a height of 1.5 meters above the ground in the cattle barn, and the temperature and humidity were automatically recorded every 30 min. The data were exported in document format. The THI was calculated using the following formula: THI = 0.8 × Tdb + RH/100 × (Tdb −14.4) + 46.4 (Zähner et al., 2004), Tdb is the dry-bulb temperature, and RH is the relative humidity. Rectal temperature and respiratory rate were measured daily at 12:00 PM. Rectal temperature was recorded using an electronic veterinary thermometer, and respiratory rate was measured by observing the abdominal movements of the cows in a lying position. Each abdominal movement cycle was considered one complete breath, and the cow was observed for three consecutive cycles.

Samples were collected on the last day of both the summer and autumn experiments. At 13:00, venous blood was collected from the coccygeal vein of the cows. The blood was allowed to stand for 30 min, then centrifuged at 3,000 rpm for 10 min at 4°C. The supernatant was collected for further analysis. The concentrations of HSP70, HSP90, SOD, T3, T4, and COR were measured using a double-antibody sandwich ELISA method. The total antioxidant capacity (T-AOC) was measured using a colorimetric assay with a reagent kit purchased from Shanghai Enzyme-linked Biotechnology Co., Ltd. DNA extraction, 16S rRNA gene amplicon sequencing and data processing.

### 2.3 DNA Extraction, 16S rDNA sequencing, and sequences analysis

Fecal samples were collected via rectal sampling and stored in cryovials, which were then placed in liquid nitrogen for preservation. These samples were used for microbiome and metabolomics analysis. The procedure was as follows: (1) DNA Extraction: Fecal DNA was extracted using a fecal DNA extraction kit (Model D3141, Guangzhou Meiji Biotechnology Co., Ltd., China). (2) DNA Quality Testing: The DNA integrity of the samples was assessed using a Nanodrop spectrophotometer (NanoDrop 2000, Thermo Fisher Scientific, United States), an agarose gel electrophoresis system (DYY-6C, Beijing Liuyi Instrument Factory, China), and a gel imaging system (Tanon-2500, Shanghai Tianen Technology Co., Ltd., China). This was to ensure that the DNA was not degraded and free from protein contamination. (3) PCR Amplification: Universal bacterial primers (341F: 5′–CCTACGGGNGGCWGCAG–3′, 806R: 5′–GGACTACHVGGGTATCTAAT–3′, amplicon length: 466 bp) were used to amplify the V3-V4 region of bacterial 16S rRNA genes. PCR was performed using a PCR machine (ETC811, Dongsheng Xinye Scientific Instrument Co., Ltd., Beijing, China). AMPure XP Beads were used for PCR product purification, and after purification, quantification was performed using a Qubit 3.0 fluorometer. (4) Library Quantification and Sequencing: The second-round PCR products were purified using AMPure XP Beads, and quantification was carried out using an ABI StepOnePlus Real-Time PCR System (Life Technologies, United States). Sequencing was performed on the Novaseq 6000 platform using the PE250 mode for pooling and sequencing. After sequencing, the raw sequences were processed using MOTHUR software. Non-redundant sequences were clustered into operational taxonomic units (OTUs) using the UPARSE software. The abundance and diversity indices of the microbiota were analyzed and compared to databases using QIIME software. Additionally, the microbial community differences among groups were analyzed.

### 2.4 Metabolomics profiling and data analysis

Hindgut samples were individually grounded with liquid nitrogen and the homogenate was resuspended with prechilled 80% methanol and 0.1% formic acid by well vortex. The samples were incubated on ice for 5 min and then were centrifuged at 15,000 g, 4°C for 20 min. Some of supernatant was diluted to final concentration containing 53% methanol by LC-MS grade water. The samples were subsequently transferred to a fresh Eppendorf tube and then were centrifuged at 15,000 g, 4°C for 20 min. Finally, the supernatant was injected into the LC-MS/MS system analysis.

The raw data files generated by UHPLC-MS/MS were processed using the Compound Discoverer 3.1 (CD3.1, Thermo Fisher) to perform peak alignment, peak picking, and quantitation for each metabolite. The main parameters were set as follows: retention time tolerance, 0.2 min; actual mass tolerance, 5 ppm; signal intensity tolerance, 30%; signal/noise ratio, 3; and minimum intensity, 100,000. After that, peak intensities were normalized to the total spectral intensity. The normalized data was used to predict the molecular formula based on additive ions, molecular ion peaks and fragment ions. And then peaks were matched with the mzCloud,^1^ Vaultand MassListdatabase to obtain the accurate qualitative and relative quantitative results. Statistical analyses were performed using the statistical software R (R version R-3.4.3), Python (Python 2.7.6 version), and CentOS (CentOS release 6.6). When data were not normally distributed, normal transformations were attempted using of area normalization method.

### 2.5 Statistical analysis

To evaluate whether the sample size (*n* = 7) provided sufficient statistical sensitivity, a *post hoc* power analysis was performed based on the observed effect sizes (Cohen’s d) for each variable. The results indicated that the indicators exhibited large to extremely large effect sizes, with statistical power values exceeding 0.80, demonstrating adequate capability to detect group differences for these traits. The physiological and biochemical indices of the cows and the alpha diversity of the hindgut microbiota were evaluated using Independent-Samples *t*-test in IBM SPSS Statistics software. The beta diversity of the hindgut microbiota was assessed using Principal Coordinate Analysis (PCoA) based on the Bray-Curtis distance algorithm, and statistical significance was tested using the Adonis test. The relative abundance differences at the phylum and genus levels were analyzed using the Wilcoxon rank-sum test. Significant differences were declared at *p* < 0.05, whereas *p* < 0.01 was considered highly significant. The results of these analyses were expressed as means and standard error of the means (SEM).

## 3 Results

### 3.1 THI and physiological parameters

The overall average daily THI was 76.87 in August and 61.63 in October, indicating markedly different environmental thermal conditions between the two experimental periods (Kim et al., 2021; Wang et al., 2025), consistent with the differences in environmental thermal load, rectal temperature (*p* < 0.01) and respiratory rate (*p* < 0.01) were significantly higher in the HS group than in the NHS group. In addition, plasma levels of HSP70, HSP90, and cortisol were significantly elevated in the HS group (*p* < 0.01), whereas T-AOC (*p* < 0.05), SOD (*p* < 0.05), T3 (*p* < 0.01), and T4 (*p* < 0.01) were significantly reduced (Table 2).

**TABLE 2:** Differential response in physiological parameters, blood parameters, for Simmental heifers under heat stress (NHS; *n* = 6) and non-heat stress (HS; *n* = 6) conditions.

Index	HS	NHS	SEM	*p*-value
**Physiological parameters**				
Respiratory rate (bpm)	49.97	21.36	2.08	0.001
Rectal temperature (°C)	38.84	38.51	0.086	0.01
**Blood parameters**				
HSP70 (ng/mL)	409.77	309.61	15.44	0.001
HSP90 (ng/mL)	236.19	180.15	5.51	0.001
T-AOC (U/mL)	0.91	1.03	0.05	0.029
SOD (pg/mL)	405.93	464.62	12.59	0.001
T3 (pmol/L)	67.41	79.92	2.59	0.001
T4 (pmol/L)	1139.76	1238.77	17.34	0.001
COR (μg/L)	202.60	171.74	4.26	0.001

Bpm, breath per minute; HSP, heat stress protein. Physiological parameters were measured daily at 12:00 PM; blood samples were collected on the last day of both the summer and autumn experiments.

### 3.2 Hindgut bacteria diversity and composition

**FIGURE 1:**
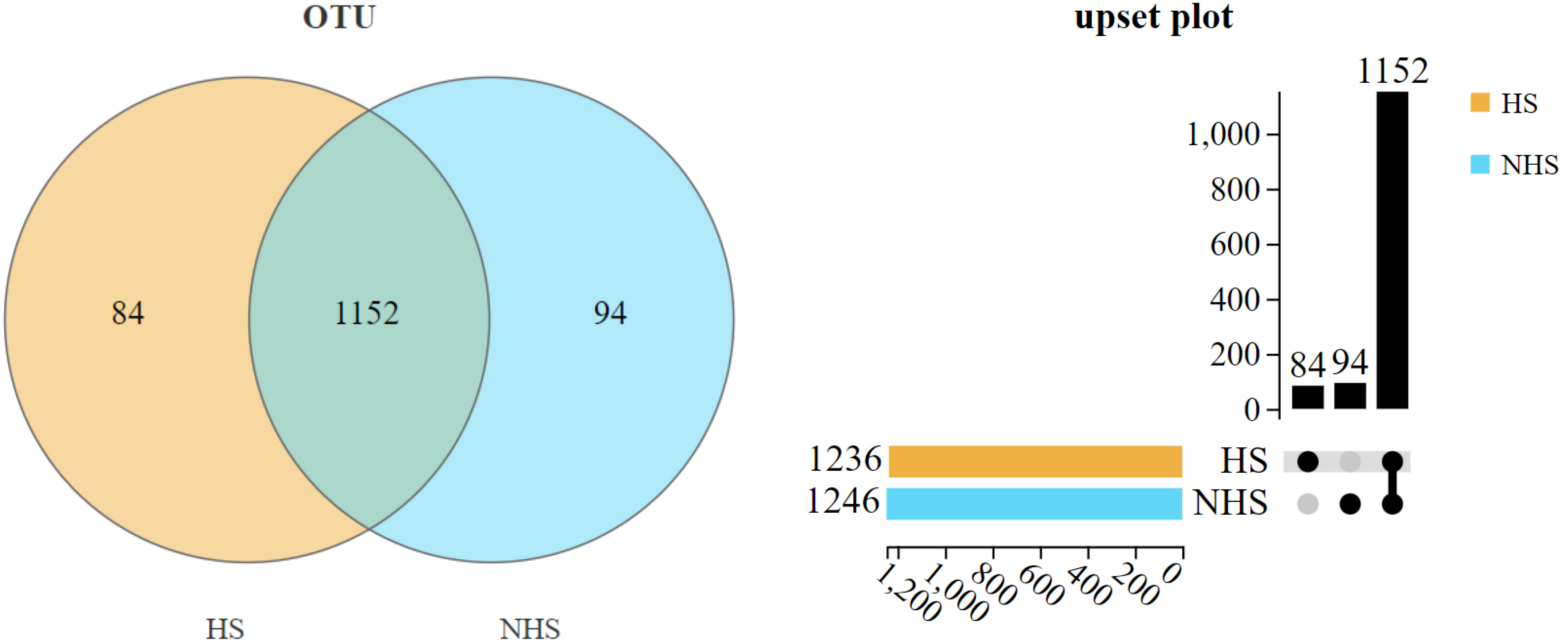
Effects of HS on OTUs of hindgut bacteria in Simmental heifers. NHS, non-heat stress; HS, heat stress.

In this experiment, the sequences were clustered at the OTU level with a similarity threshold of 97%, yielding the following results: As shown in Figure 1, the VN diagram and Upset plot visually represent the shared and similarity status of OTUs between the two groups. From the VN diagram, it can be observed that the HS group and NHS group possess 1,236 and 1,246 OTUs, respectively, with 1,152 OTUs being shared. Specifically, the HS group has 84 unique OTUs, while the NHS group has 94 unique OTUs.

**FIGURE 2:**
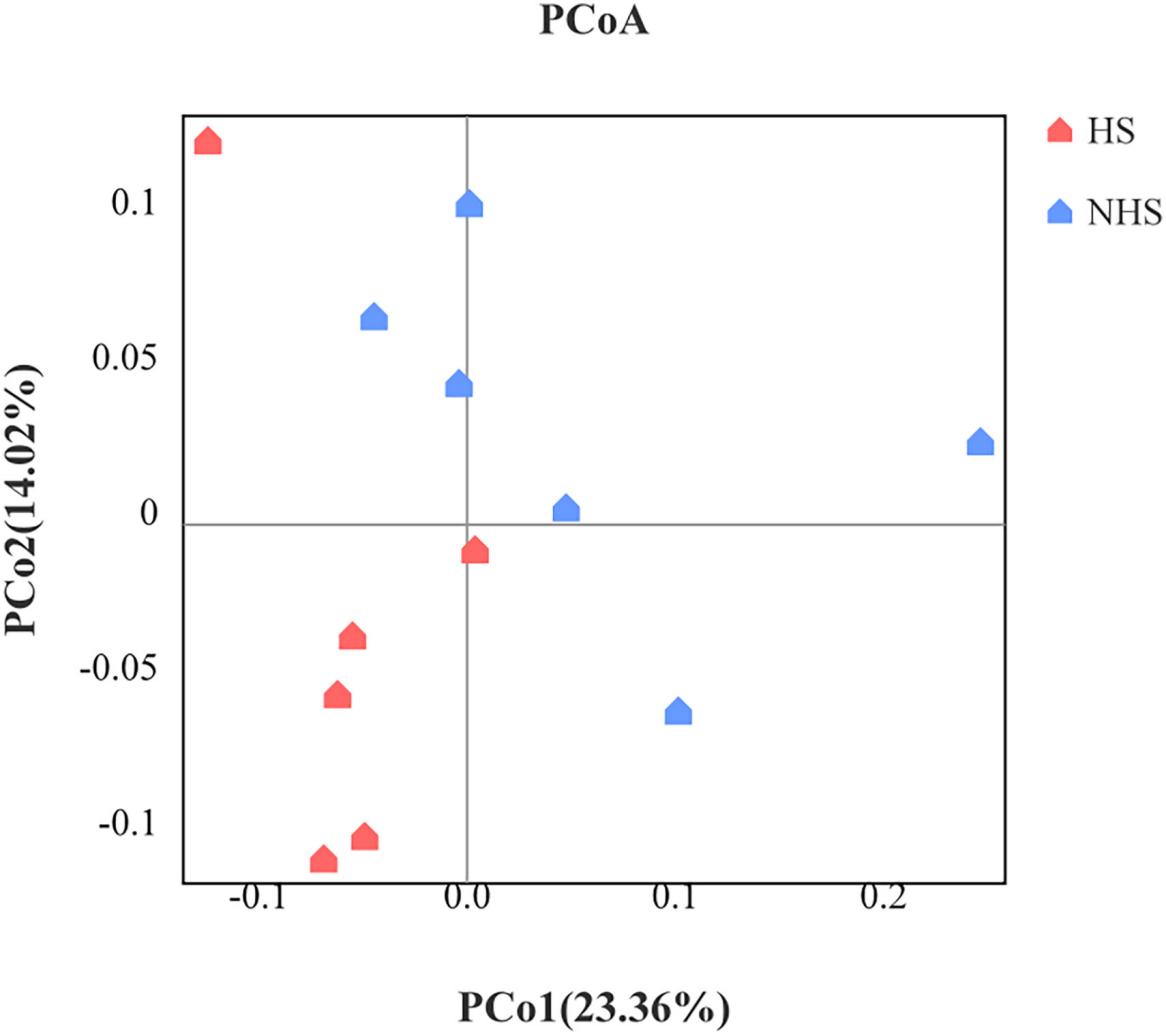
Effects of HS on richness and diversity of hindgut bacteria in Simmental heifers. NHS, non- heat stress; HS, heat stress.

At the 97% similarity threshold, the total OTUs identified were subjected to alpha diversity index statistical analysis (Figure 2). From the figure, it can be observed that heat stress did not significantly affect the alpha diversity of the hindgut microbiota in Simmental heifers, as indicated by the Shannon, Ace, Sobs, Simpson, Chao1, and Good Coverage indices (*P* > 0.05). Heat stress did have some impact on the diversity of the hindgut microbiota, but no significant effect on its richness was observed.

**FIGURE 3:**
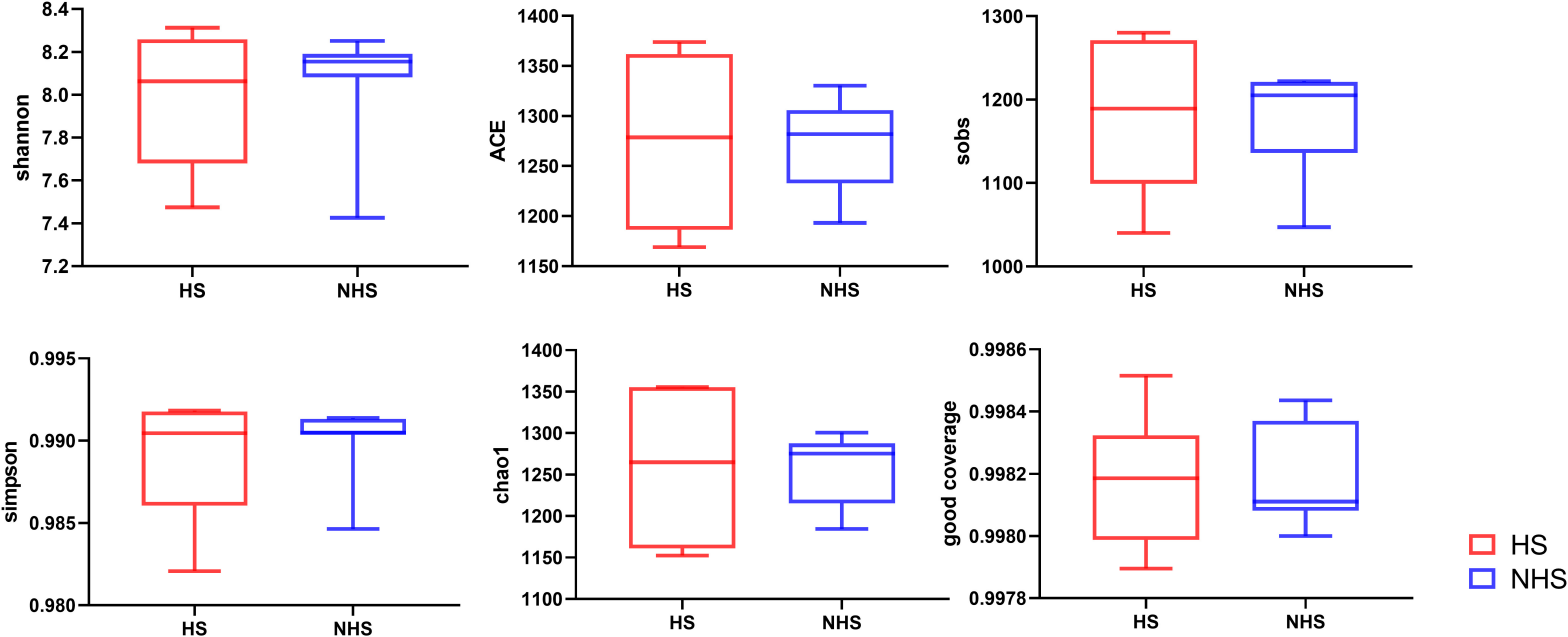
Beta diversity of hindgut microbiota using principal co-ordinates analysis. NHS, non-heat stress condition; HS, heat stress condition.

PCoA was performed using the Bray-Curtis distance algorithm. The principal coordinates were PCo1 (23.36%) and PCo2 (14.02%), with a total contribution of 37.38%, which comprehensively reflects the information of the samples. A clear separation between the two groups was observed, indicating that there were differences in the hindgut microbiota between the HS and NHS groups. This suggests that heat stress has an impact on the development of the hindgut microbiota in Simmental heifers. The inter-group differences were further tested using the Adonis test, with an R2 of 0.1109 and *P* = 0.031, indicating that the Beta diversity of the hindgut microbiota between the HS and NHS groups was significantly different (*P* < 0.05) (Figure 3).

**FIGURE 4:**
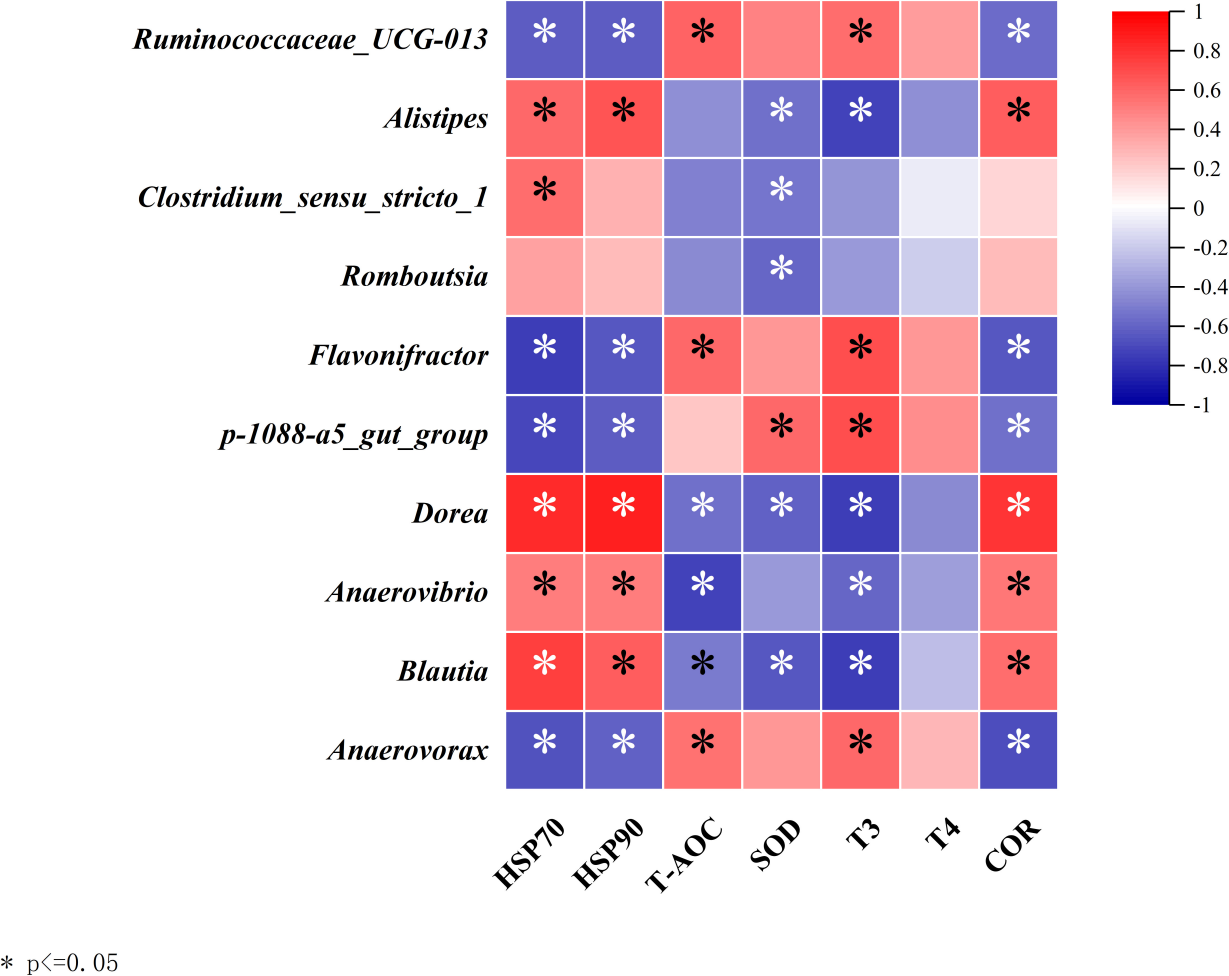
Effects of HS on the phylum-level hindgut microbiota in Simmental heifers. NHS, non-heat stress condition; HS, heat stress condition.

To further explore the impact of heat stress on the composition of the hindgut microbiota at different taxonomic levels, the microbiota was identified at the phylum level (Figure 4). At the phylum level, significant differences were observed between the HS and NHS groups in the relative abundance of Proteobacteria, Planctomycetes, and Lentisphaerae (*P* < 0.05).

**FIGURE 5:**
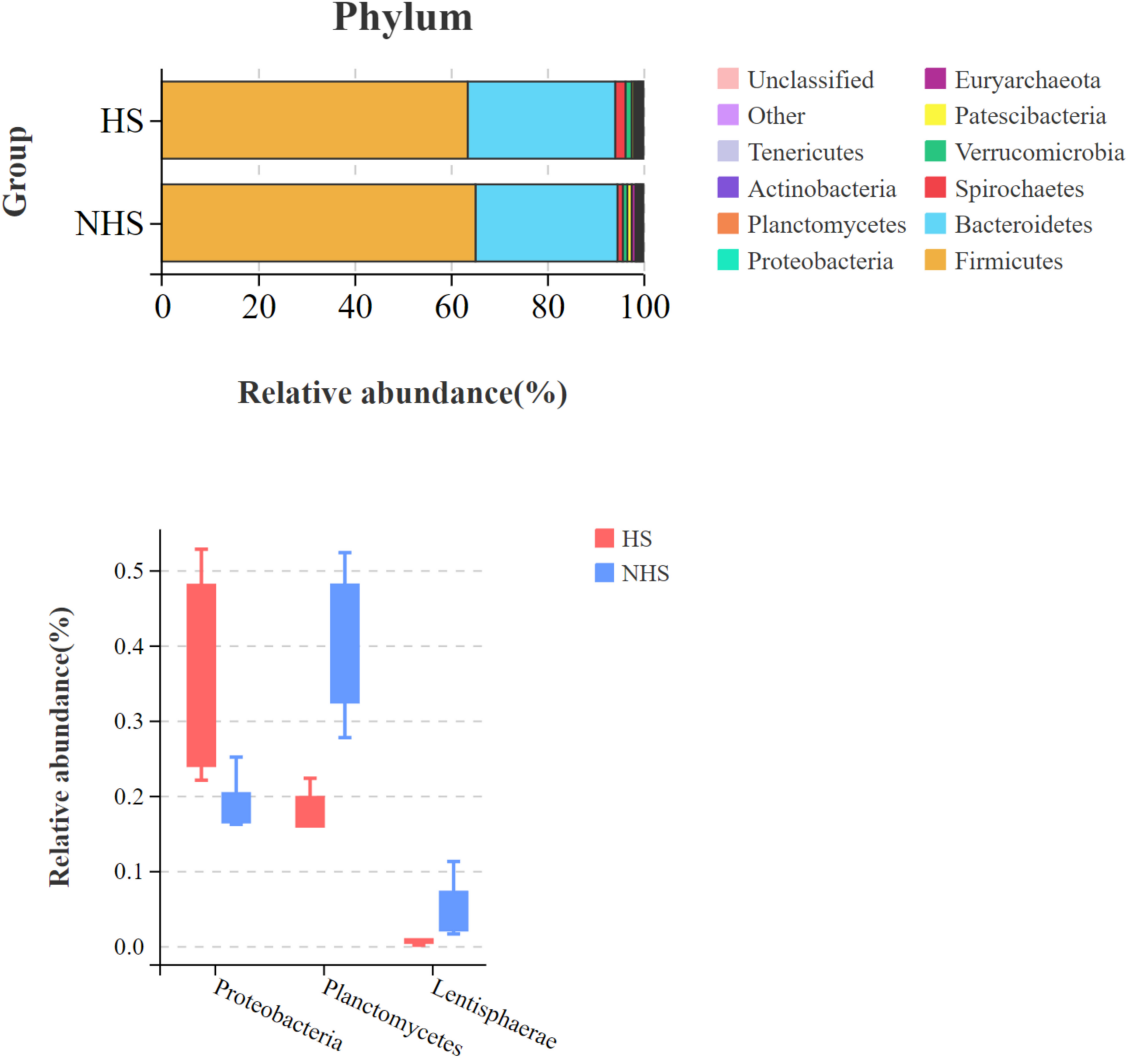
Effects of HS on the Genus-Level hindgut microbiota in Simmental heifers. NHS, non-heat stress condition; HS, heat stress condition.

At the genus level, the dominant genera in the hindgut microbiota of Simmental heifers in the HS and NHS groups were Ruminococcaceae_UCG-005, Rikenellaceae_RC9_gut_group, Eubacterium_coprostanoligenes_group, and Ruminococcaceae_UCG-010. Differential statistical analysis of the genus-level microbiota in the hindgut of Simmental heifers in the HS and NHS groups was performed using the Wilcoxon rank-sum test, with the results shown in Figure 5 and Table 3. A total of 10 genera were identified with significant differences, including Ruminococcaceae_UCG-013, Alistipes, Clostridium_sensu_stricto_1, Romboutsia, Flavonifractor, p-1088-a5_gut_group, Dorea, Anaerovibrio, Blautia, and Anaerovorax (*P* < 0.05).

**TABLE 3:** Differences microbes of Simmental heifers intestines in the HS and NHS group at the genus level.

Items	HS	NHS	*P*-value
Ruminococcaceae_UCG-013	3.369 ± 0.19	4.348 ± 0.25	0.026
Alistipes	3.782 ± 0.18	3.191 ± 0.22	0.041
Clostridium_sensu_ stricto_1	1.502 ± 0.33	0.652 ± 0.13	0.026
Romboutsia	1.006 ± 0.11	0.724 ± 0.11	0.041
Flavonifractor	0.383 ± 0.03	0.563 ± 0.02	0.009
p-1088-a5_gut_group	0.136 ± 0.03	0.382 ± 0.06	0.002
Dorea	0.194 ± 0.01	0.094 ± 0.02	0.002
Anaerovibrio	0.190 ± 0.05	0.060 ± 0.03	0.041
Blautia	0.136 ± 0.02	0.076 ± 0.01	0.009
Anaerovorax	0.067 ± 0.01	0.119 ± 0.01	0.015

The data were analyzed by Wilcoxon rank sum test and Tukey multiple test. *P* < 0.05 was considered as significant difference and marked with different lowercase letters.

**FIGURE 6:**
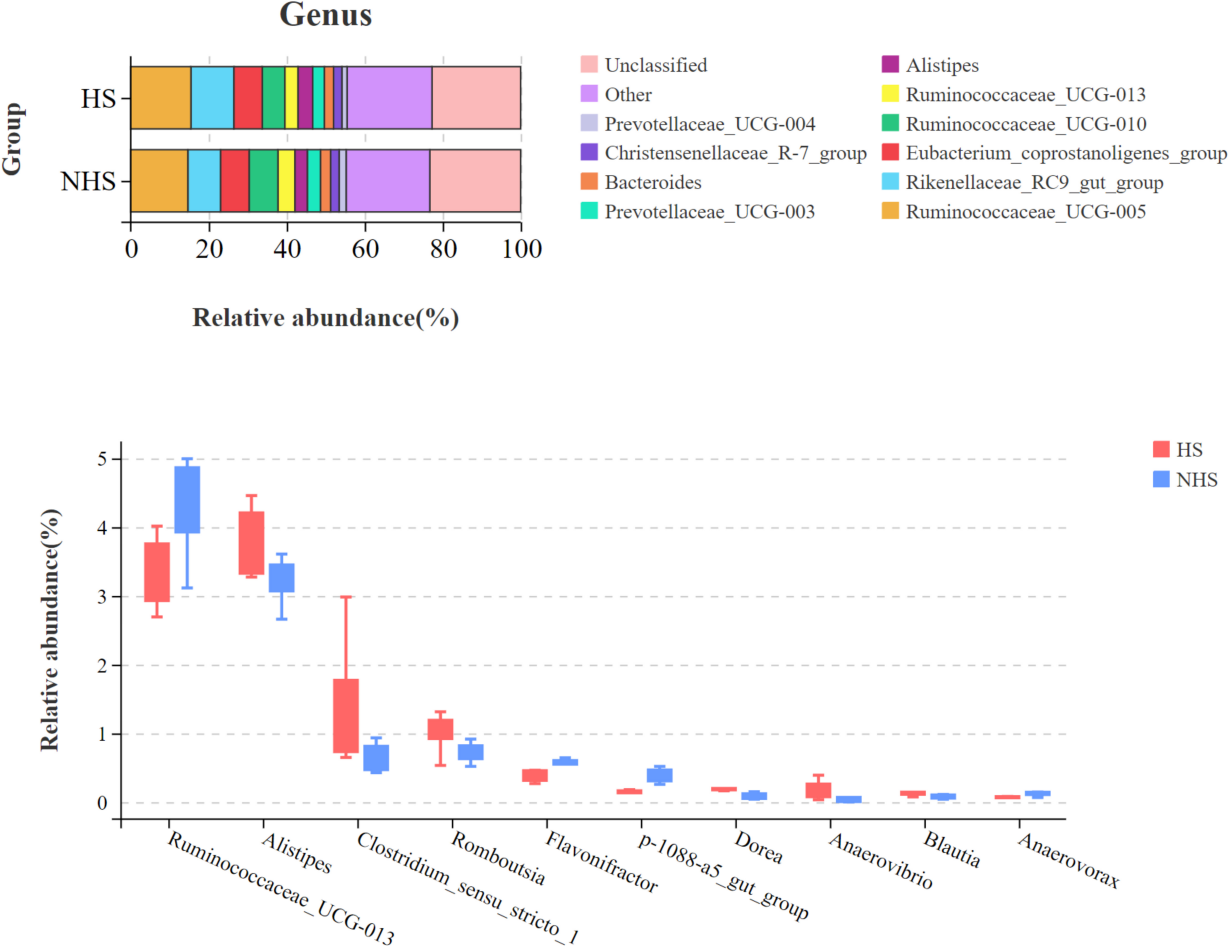
Efect of heat stress on correlation analysis between hindgut bacteria and blood parameters assessed by Spearman correlation analyses. NHS, non-heat stress condition: HS, heat stresscondition. Red indicates a positive correlation; the blue indicates a negative correlation; asterisksdenote significant differences at *p* < 0.05.

To further investigate whether the changes in the hindgut microbiota of Simmental heifers due to heat stress are associated with relevant blood parameters, Spearman correlation analysis was performed between the significantly different genera and heat stress-related blood indicators (Figure 6). The results indicated that genera such as Ruminococcaceae_UCG-013, Alistipes, Clostridium_sensu_stricto_1, and Romboutsia showed a certain degree of correlation with the blood parameters.

### 3.3 Identification of different metabolites and metabolic pathways

**FIGURE 7:**
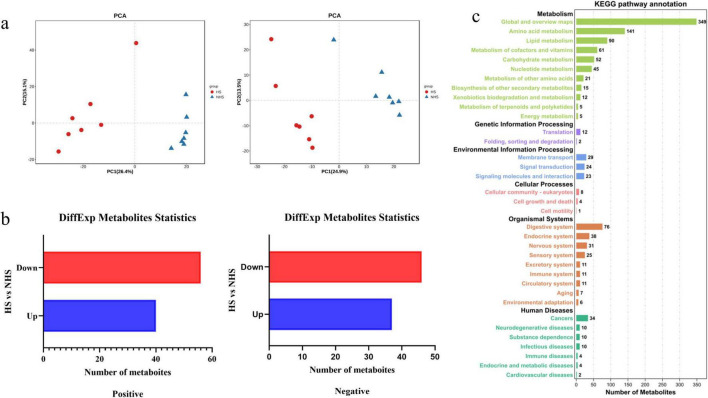
Effects of metabolites in hindgut of Simmental heifers under non-heat stress and heat stress conditions. **(a)** PCA analysis of HS group and NHS group **(b)** Differential metabolites between HS and NHS groups **(c)** Differential metabolites between HS and NHS groups. NHS, non-heat stress condition; HS, heat stress condition.

The PCA analysis of LC-MS metabolic profiles of hindgut samples showed a separation between the NHS and HS groups (Figure 7). Compared to the NHS group, 96 differential metabolites were identified in the positive ion mode in the HS group, with 56 metabolites being downregulated and 40 metabolites being upregulated. In the negative ion mode, 83 differential metabolites were identified, of which 46 were downregulated and 37 were upregulated. The selected metabolites were annotated (Figure 7). The functional differences in metabolites between the HS and NHS groups were primarily concentrated in the following metabolic pathways: Global and overview maps, Amino acid metabolism, Lipid metabolism, Metabolism of cofactors and vitamins, Carbohydrate metabolism, Nucleotide metabolism, and Metabolism of other amino acids. Enrichment analysis of the differential metabolites revealed seven metabolic pathways, namely Pyrimidine metabolism, Biosynthesis of unsaturated fatty acids, Amino sugar and nucleotide sugar metabolism, Fatty acid biosynthesis, Propanoate metabolism, Inositol phosphate metabolism, and Beta-alanine metabolism.

## 4 Discussion

Previous studies have shown that exposure to high THI conditions is associated with a range of physiological stress responses in cattle, although the specific THI level at which these responses occur may vary across breeds and physiological states. As the ambient temperature rises, cattle can regulate their body temperature through their thermoregulatory systems to maintain homeostasis. Once the threshold is surpassed, excess heat accumulates within the body, and physiological parameters begin to change. The endocrine system is an important mechanism for regulating environmental stress and metabolic growth, playing a role in stimulating or inhibiting physiological activities. The endocrine system does not directly regulate internal changes but rather influences the stress response by secreting hormones (Bongiovanni et al., 2018). Heat Shock Proteins (HSPs) are a series of proteins generated under stress, particularly under high-temperature conditions (Sonna et al., 2002). Among them, HSP70 is one of the most characteristic heat shock protein families, composed of highly conserved stress proteins, and it is expressed during stress responses, playing a crucial role in stress tolerance and adaptation to environmental stress (Banerjee et al., 2014). In Mishra’s study, heat stress significantly increased the expression of HSP70 in the serum of buffaloes (Mishra et al., 2011). In this experiment, compared to the NHS group, the blood of the HS group Simmental heifers showed a significant increase in HSP70 levels. Furthermore, Spearman correlation analysis demonstrated that both HSP70 and HSP90 were significantly correlated with THI as well as multiple heat stress–related physiological and biochemical indicators, supporting the use of THI as an environmental descriptor rather than a definitive diagnostic threshold (Supplementary Figure S1).

Superoxide dismutase (SOD) is a peroxidase enzyme widely distributed in the body, playing a crucial role in eliminating free radicals, maintaining oxidative balance, and protecting against oxidative damage. SOD is capable of neutralizing free radicals in the body (Fujii et al., 2022). Previous studies on bovine liver injury have shown that SOD activity is reduced in damaged liver tissue compared with healthy liver, with more severe injury being associated with a further decline in SOD activity. In addition, Malondialdehyde (MDA) levels were negatively correlated with SOD and glutathione peroxidase (GPX) activities, suggesting that a reduction in antioxidant capacity may contribute to increased free radical accumulation and enhanced lipid peroxidation (Bahrami et al., 2014). In this experiment, the levels of SOD and total antioxidant capacity (T-AOC) were significantly lower in the blood of the HS group Simmental heifers. This could be due to the high summer temperatures, which led to the production of a large number of free radicals, disrupting oxidative balance. As a result, prolonged exposure to high temperatures depleted the body’s antioxidants (Surai et al., 2019), leading to oxidative damage. Thyroid hormones, usually referring to T3 and T4, are important indicators of nutritional metabolism and play a crucial role in animal growth and reproduction (Todini, 2007). Thyroid hormones promote the breakdown and oxidation of sugars, fats, and proteins, thereby increasing the body’s heat production and oxygen consumption. During heat stress, in order to reduce metabolic heat production and maintain thermal balance, the hypothalamic-pituitary-thyroid axis responds to temperature stimuli by decreasing thyroid hormone secretion (Pereira et al., 2008). In this experiment, under high summer temperatures, the levels of T3 and T4 in the serum of Simmental heifers were significantly reduced, consistent with previous conclusions. At the same time, heat stress led to a significant increase in cortisol (COR) levels. COR, a glucocorticoid secreted by the adrenal glands under stress, plays an important role in the body’s thermal regulation. Under acute heat stress conditions, COR concentration in the serum increases (Gross and Siegel, 1973).

For animals, the gut microbiota is the result of long-term evolution and adaptation, forming a mutually dependent and symbiotic system within the intestines. The diversity of the gut microbiota is an important indicator of gut richness and the maintenance of microbiota stability. Microorganisms in the gut can participate in the host’s physiological activities through various pathways. The gut microbiota forms a barrier with the intestinal epithelial cells, helping to resist pathogen invasion and maintain the stability of the gut environment (Sansonetti, 2004). In ruminants, the gut microbiota interacts with the host and participates in the host’s nutritional metabolism and development in multiple ways. The gut microbiota extracts nutrients from the host’s diet, regulates fat storage, stimulates the metabolism of intestinal epithelial cells, and plays an important role in guiding the maturation of the immune system (Pickard et al., 2017). Furthermore, multiple metabolic pathways involve the gut microbiota, which, through signal transduction, regulates the activity of various tissues and organs, thus having a lasting impact on the production performance and health of ruminants, such as short-chain fatty acid metabolism, bile acid metabolism, and polyphenol metabolism (O’Hara et al., 2020; Sanz-Fernandez et al., 2020). Simultaneously, the gut microbiota can break down and digest non-digestible carbohydrates like cellulose and hemicellulose from the diet, forming a mutualistic symbiosis with the host and improving nutrient utilization efficiency in ruminants. Relevant studies have pointed out that heat stress reduces the richness and diversity of the gut microbiota, leading to dysbiosis (Rostagno, 2020). In this study, the impact of heat stress on the alpha diversity of the gut microbiota in Simmental cattle was non-significant, as heat stress induced alterations in the compositional structure of the gut microbial community without substantially changing the species richness. Research has concluded that microbial dysbiosis caused by metabolic disorders is typically accompanied by an increase in the relative abundance of Proteobacteria. The author also noted that the abundance of Proteobacteria represents an unstable microbial community and metabolic disorder. In dysbiotic intestines, the adaptability of Proteobacteria gradually increases, making it the dominant phylum (Shin et al., 2015). In this experiment, the relative abundance of Proteobacteria in the hindgut of the HS group Simmental heifers was significantly increased, while the relative abundance of Firmicutes showed a decreasing trend. Heat stress led to dysbiosis in the hindgut microbiota of Simmental heifers, with Firmicutes and Proteobacteria forming a competitive relationship.

At the genus level, the dominant genus is Ruminococcus, which belongs to the Firmicutes phylum. Ruminococcus is one of the earliest discovered gut microorganisms in humans and plays an important role in intestinal metabolism. It has a strong ability to digest resistant starch and can obtain nutrients by breaking down cellulose from the host’s digestive organs. It is considered a beneficial bacterium (La Reau and Suen, 2018). In this experiment, heat stress significantly reduced the relative abundance of Ruminococcaceae_UCG-013, indicating that heat stress affected the breakdown of cellulose in the gut of Simmental heifers, thereby reducing nutrient absorption.

Alistipes is a pathogenic bacterium. Related studies have shown that the enrichment of Alistipes in the intestines of mice fed a high-fat diet leads to the conclusion that Alistipes, as a pathogenic bacterium, directly causes the proliferation of colon cells and severely damages the gut barrier function. Other studies have also shown that Alistipes converts lactate into other short-chain fatty acids, such as formic acid, acetic acid, and propionic acid. When the levels of these fatty acids exceed a certain threshold, they can damage the intestinal walls, leading to increased intestinal permeability, inflammation, and even autoimmune diseases (Igarashi et al., 2016). In this experiment, the relative abundance of Alistipes in the HS group was significantly higher than in the NHS group, suggesting that the heat stress in summer may have caused intestinal inflammation in Simmental heifers, further leading to gut microbiota dysbiosis.

In this experiment, heat stress significantly increased the relative abundance of Clostridium_sensu_stricto_1, indicating that the abundance of Clostridium_sensu_stricto_1 is closely related to inflammatory responses in the gut. Flavonifractor, which belongs to Ruminococcus, plays an important role in butyrate production (Rodriguez-Castaño et al., 2020). The addition of butyrate to the diet can improve the feed conversion rate in ruminants. In this study, the relative abundance of Flavonifractor in the HS group was significantly reduced compared to the NHS group, suggesting that heat stress may have affected the digestion and absorption of nutrients by the gut microbiota in Simmental heifers. Research has confirmed that prolonged high temperatures in summer lead to abnormal increases in plasma levels of very low-density lipoprotein (VLDL) and cholesterol in livestock, along with decreased enzyme activity and impaired liver fat synthesis, resulting in lipid metabolism disorders (Hall et al., 2000). A study confirmed that Dorea can prevent obesity and liver fat degeneration induced by a high-fat diet and plays a significant role in improving lipid and glucose metabolism (Maqoud et al., 2024). In this experiment, heat stress led to gut microbiota dysbiosis in Simmental heifers, and the relative abundance of Dorea significantly increased. When the heat stress phase ended, the gut microbiota gradually returned to normal, with the relative abundance of Dorea returning to normal as well, indicating that Dorea plays a regulatory role in restoring gut microbiota balance disturbed by heat stress. Susanna’s research on the gut microbiota of herbivorous and omnivorous beef cattle found that the relative abundance of Anaerovorax was significantly higher in the gut microbiota of omnivorous cattle than in herbivorous cattle (Lau et al., 2018). These genera participate in the conversion of putrescine into butyrate and may provide additional energy to ruminants through the production of short-chain fatty acids (Matthies et al., 2000; Ueki et al., 2018). In this study, after the heat stress phase ended, the relative abundance of Anaerovorax significantly increased, providing additional energy for the growth of Simmental heifers. In conclusion, under the influence of heat stress, significant differences in the gut microbiota of both HS and NHS group Simmental heifers were observed, primarily manifested in inflammation responses, nutrient absorption, and energy metabolism.

Alistipes and Romboutsia primarily function in the gut in relation to inflammation. During the heat stress phase, prolonged high temperatures due to the hot climate induce an inflammatory response in Simmental cattle. The abundance of Alistipes and Romboutsia increases, severely harming the health of Simmental cattle. Heat shock proteins, as repair proteins, decompose damaged cells and recycle raw materials for protein synthesis, ensuring the smooth operation of cellular physiological processes. In this experiment, the results showed a significant positive correlation between heat shock proteins and Alistipes, Clostridium_sensu_stricto_1, and Romboutsia, indicating that heat shock proteins are involved in repairing damaged cells during the heat stress phase. Once heat stress subsides, inflammation in Simmental cattle disappears, the abundance of harmful bacteria in the gut decreases, and the synthesis and repair functions of heat shock proteins gradually stabilize. Ruminococcaceae_UCG-013, Flavonifractor, and Anaerovorax are categorized according to their functions described earlier, with their main role being the absorption and metabolism of nutrients. COR, a glucocorticoid secreted by the adrenal glands, is a stress hormone. When environmental changes exceed the tolerance of the body, COR is secreted in large amounts. Under heat stress conditions, COR induces immune responses, leading to the excessive oxidation of amino acids in the animal’s body rather than their conversion into protein deposits. This significantly affects the production performance of livestock and poultry. Ruminococcaceae_UCG-013, Flavonifractor, and Anaerovorax, as beneficial microbiota related to nutrient absorption, show a significant negative correlation with COR. These beneficial microbiota are impaired under heat stress, and at this time, COR’s anti-inflammatory effects play a regulatory role in the inflammation caused by heat stress. COR achieves this by enhancing the activity of protein deacetylases, which may also reduce the levels of pro-inflammatory factors and enhance the activity of nuclear factor kappa-light-chain-enhancer of activated B cells (NF-kB). Additionally, by inhibiting the transcription of pro-inflammatory nuclear factor activation proteins, COR suppresses the expression of inflammation-related genes, thereby regulating the inflammatory process. This leads to a reduction in inflammation and the restoration of the abundance of Ruminococcaceae_UCG-013, Flavonifractor and Anaerovorax, ultimately restoring the normal nutritional metabolism function of the gut microbiota in Simmental cattle.

In this experiment, the impact of heat stress on the hindgut metabolism of Simmental cattle was primarily concentrated on pyrimidine metabolism, biosynthesis of unsaturated fatty acids, amino sugar and nucleotide sugar metabolism, as well as fatty acid biosynthesis and propanoate metabolism. Heat stress significantly decreased the levels of uridine, uracil, and thymidine, which are involved in pyrimidine metabolism. Pyrimidine metabolism is an important pathway for maintaining the stability of the intracellular pyrimidine pool. Additionally, pyrimidines undergo degradation, producing various precursor molecules for vitamins, pantothenic acid, and other biomolecules (Tiedje et al., 2010). In this study, the contents of uracil, thymidine, and cytosine in the HS group were significantly altered, indicating that pyrimidine metabolism is involved in the body’s stress response. Glucosamine is an important functional monosaccharide, and N-acetylglucosamine is a product of the acetylation of glucosamine. Both glucosamine and N-acetylglucosamine play critical roles in biological growth processes (Yadav et al., 2011). In this experiment, significant differences were observed in the amino sugar and nucleotide sugar metabolism pathways between the HS and NHS groups. Heat stress led to a significant downregulation of D-glucosamine and N-acetylglucosamine, suggesting that heat stress may affect the growth and development of Simmental cattle. Ketone bodies are intermediate products of fatty acid oxidation, and when the body is in a state of hunger or low blood glucose levels, it synthesizes large amounts of acetone to provide energy (Kubera et al., 2014). Acetone metabolism is part of propanoate metabolism. In this study, heat stress led to a downregulation of acetoacetate synthesis in propanoate metabolism, further reducing the production of acetone. This suggests that heat stress may impair energy metabolism, and the body was unable to compensate for the reduction in productivity caused by heat stress. Related research indicates that heat stress accelerates lipid metabolism, and prolonged heat stress inhibits the breakdown of fat tissue (Teyssier et al., 2023), Stearic acid, through β-oxidation, can generate large amounts of ATP, much more than the ATP generated by glucose metabolism. In this study, heat stress led to a decrease in the concentration of stearic acid in the hindgut of Simmental cattle, indicating that during the heat stress phase, the β-oxidation of stearic acid helps glucose metabolism to generate energy, meeting the body’s needs.

## 5 Conclusion

High temperatures induce heat stress responses in Simmental cattle, leading to an increase in their respiratory rate and rectal temperature, while also causing changes in heat stress-related blood indicators. These changes are reflected in impaired antioxidant capacity, increased levels of heat shock proteins and COR, and abnormal alterations in thyroid hormones. Heat stress is associated with alterations in the gut microbiota of Simmental cattle, with the main effects observed in nutrient absorption metabolism and inflammation. Heat stress results in a reduction in the abundance of certain beneficial bacteria and an increase in harmful bacteria, which alters the small molecule metabolites in the gut of Simmental cattle. These changes in metabolites involve pyrimidine metabolism, biosynthesis of unsaturated fatty acids, amino sugar and nucleotide sugar metabolism, fatty acid biosynthesis, and propanoate metabolism. The findings of this study provide new insights into understanding the effects of heat stress on gut microbiota and its metabolites. Further research is needed to validate the results of this study.

## Data Availability

The raw data generated in this study have been deposited in the NCBI Sequence Read Archive (SRA) under accession number PRJNA1397550.

## Footnotes

1
https://www.mzcloud.org/mz

## Supplementary material

Supplementary Figure S1 Spearman correlation analysis between heat shock proteins and environmental and biochemical indicators of heat stress. Orange indicates a positive correlation; blue indicates a negative correlation; asterisks denote significant differences at *p* < 0.05.
