# Supplementary figures and images for: The effect of heat stress on the hindgut microbiota and metabolites of Simmental heifers
Source: Front Microbiol. 2026 Jan 20;16:1724640. doi: 10.3389/fmicb.2025.1724640 (PMC12865302; doi:10.3389/fmicb.2025.1724640)

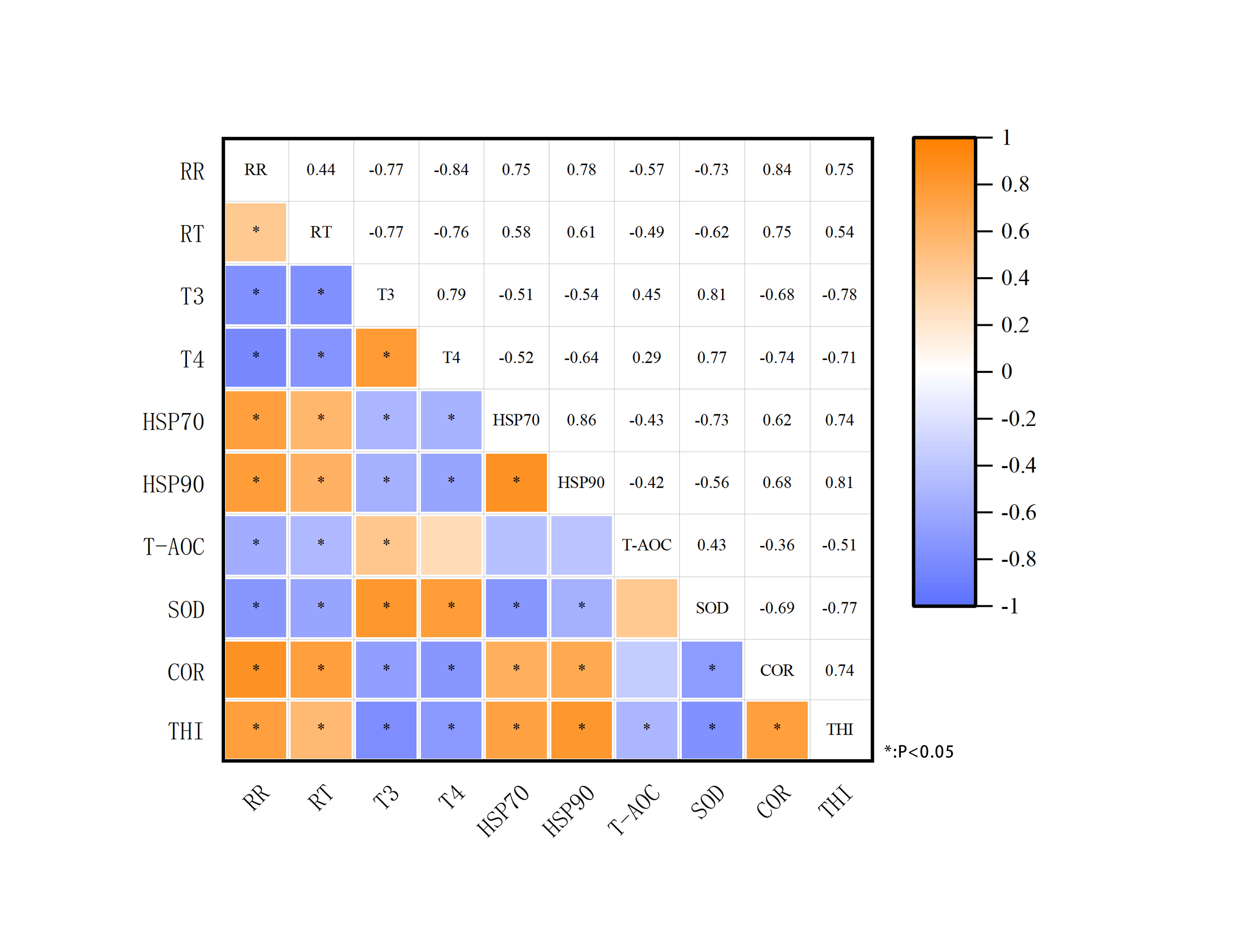

Supplement: Supplementary Figure S1 — Spearman correlation analysis between heat shock proteins and environmental and biochemical indicators of heat stress. Orange indicates a positive correlation; blue indicates a negative correlation; asterisks denote significant differences at p < 0.05. [file Image_1.tif]
